# Factors Affecting Participation in Leisure Activities in Patients after Breast Cancer Surgery

**DOI:** 10.3390/healthcare9081078

**Published:** 2021-08-22

**Authors:** Yoshiteru Akezaki, Eiji Nakata, Masato Kikuuchi, Ritsuko Tominaga, Hideaki Kurokawa, Masaki Okamoto, Makiko Hamada, Kenjiro Aogi, Shozo Ohsumi, Shinsuke Sugihara

**Affiliations:** 1 Division of Physical Therapy, Kochi Professional University of Rehabilitation, Kochi 781-1102, Japan; akezakiteru@yahoo.co.jp; 2 Department of Orthopaedic Surgery, Okayama University Hospital, Okayama 700-8558, Japan; 3 Department of Rehabilitation Medicine, National Hospital Organization Shikoku Cancer Center, Ehime 791-0280, Japan; kikuuchi.masato.tu@mail.hosp.go.jp (M.K.); tominaga.ritsuko.fk@mail.hosp.go.jp (R.T.); kurokawa.hideaki.ac@mail.hosp.go.jp (H.K.); okamoto.masaki.cb@mail.hosp.go.jp (M.O.); sugihara.shinsuke.rk@mail.hosp.go.jp (S.S.); 4 Department of Rehabilitation Medicine, Higashi Tokushima Medical Center, Tokushima 779-0193, Japan; hamada.makiko.wu@mail.hosp.go.jp; 5 Department of Breast Oncology, National Hospital Organization Shikoku Cancer Center, Ehime 791-0280, Japan; aogi.kenjiro.zx@mail.hosp.go.jp (K.A.); osumi.shozo.ur@mail.hosp.go.jp (S.O.)

**Keywords:** breast cancer, leisure, surgery, rehabilitation, factor

## Abstract

Background: The purpose of this study was to investigate the factors related to patient’s participation in leisure activity in breast cancer patients with axillary lymph node dissection at 3 months after surgery. Methods: In total, 160 women who were employed before their surgery were evaluated. Age, body mass index (BMI), employment, level of lymph node dissection, marital status, children, coresident household members, preoperative chemotherapy, postoperative chemotherapy, postoperative hormonal therapy, postoperative radiotherapy, shoulder range of motion test, upper limb function, quality of life, and patient’s participation in leisure activity were evaluated. Results: Patients who undertook leisure activities constituted the leisure activity group, and patients who did not constituted the non-leisure activity group. Global health status, emotional function, social function, and dyspnea were significantly different between the leisure activity group and the non-leisure activity group at 3 months after surgery (*p* < 0.05). Regarding factors that affected participation in leisure activities, logistic regression analysis showed that only participation in leisure activities before surgery was significantly associated with participation in leisure activities at 3 months after surgery (*p* < 0.05). Conclusion: Patients who did not participate in leisure activities prior to surgery were unlikely to participate 3 months after surgery and thus require intervention to encourage their involvement.

## 1. Introduction

Breast cancer is the most frequent cancer type among women [1]. Advances in medicine and technology enable early diagnosis of breast cancer and better treatment options, improving survival rates. Breast cancer treatment modalities include surgery, chemotherapy, and radiation therapy. However, surgery can cause pain, decreased muscle strength, reduced range of motion (ROM) of the shoulder, lymphedema, axillary web syndrome, and psychological problems [2,3,4,5,6,7].

Although not necessarily the case in patients in the prediagnosis phase, the main goal of many cancer patients is to return to a level of “normalcy” after the completion of their treatment [8]. In postoperative patients with breast cancer, in addition to improving upper limb function, a return to daily life may lead to improved quality of life (QOL). Daily life includes participation in leisure activities, which may mitigate disease-induced stress and reduce feelings of isolation and loneliness [9]. Positively experienced developments in hobbies after diagnosis is shown to reduce deaths from breast cancer [10]. Patients with hobbies lived longer than those without, with an increased number of hobbies reducing the risk of death and curability depending on treatment [9]. Although arm morbidity has been reported as a factor influencing difficulties with patient’s participation in leisure activity and negative changes in patient’s participation in leisure activity [11], factors influencing postoperative patient’s participation in leisure activity remain unclear. Identifying factors that influence patient’s participation in leisure activity after surgery will help in implementing interventions that promote patient’s participation in leisure activity.

The purpose of this study was to investigate the factors related to patient’s participation in leisure activity in breast cancer patients with axillary lymph node dissection at 3 months after surgery.

## 2. Methods

### 2.1. Patients and Methods

Of the breast cancer patients who underwent mastectomy with lymph node dissection at our institution from November 2013 to December 2016, 237 patients who were referred to the Department of Rehabilitation Medicine had available data of factors investigated for 3 months. Among them, 160 patients with available data of participation in preoperative and 3-month postoperative leisure activities were selected for statistical analyses.

Age, body mass index (BMI), employment (yes/no), level of lymph node dissection (level 1 or level 2 and higher), marital status (married: yes or no), children (yes or no), coresident household members (yes or no), preoperative chemotherapy (yes or no), postoperative chemotherapy (yes or no), postoperative hormonal therapy (yes or no), postoperative radiotherapy (yes or no), shoulder ROM (degrees), upper limb function (Disabilities of the Arm, Shoulder and Hand (DASH)) (scores), patient’s participation in leisure activity before surgery (yes/no), QOL (The European Organization for Research and Treatment of Cancer (EORTC) QLQ-C30) (scores), and patient’s participation in leisure activity after surgery were evaluated.

### 2.2. Rehabilitation Program

As part of preoperative rehabilitation during hospitalization, we used a digital versatile disc (DVD) to demonstrate exercises for the upper limbs. Postoperative rehabilitation started on the first and second days after surgery, and exercise was performed beyond the elbow joint. From the third postoperative day to the day of drain withdrawal, ROM exercise of the upper limbs was performed within 90 degrees of flexion and abduction of the shoulder joint. After removal of the drain, shoulder ROM was not restricted, and upper limb movements and activities of daily living (ADL) were adjusted according to the degree of pain. Patients were instructed to self-train using a DVD at home at least once a day after discharge. At 1, 2, and 3 months after surgery, an occupational therapist and a physical therapist evaluated the patient’s upper limb function, QOL, exercise status, and participation in leisure activities. Then, we instructed on exercise, ADL, and leisure activities according to the patient’s evaluation results.

### 2.3. Shoulder Range of Motion Test

An occupational therapist and a physical therapist assessed shoulder flexion and performed an abduction active shoulder ROM test (ROM-T) with a goniometer. The shoulder ROM used the evaluation results at 3 months after surgery.

### 2.4. Disabilities of the Arm, Shoulder and Hand

The DASH questionnaire was used to evaluate the functional status of the upper extremities. DASH consists of a 30-item disability/symptom scale for the patient’s health status during the preceding week [12] and used evaluation results at 3 months after surgery.

### 2.5. Quality of Life Assessment

The EORTC QLQ-C30 was organized into global health status, five functional scales (physical, role, emotional, cognitive, and social), three symptom scales (fatigue, nausea/vomiting, and pain), single items assessing additional symptoms (dyspnea, insomnia, appetite loss, constipation, and diarrhea), and perceived financial difficulties [13]. Higher scores for global health status and for functioning indicate better health. Higher scores for symptoms indicate worse health. The EORTC QLQ-C30 measurements used evaluation results at 3 months after surgery.

### 2.6. Leisure Activity Participation Assessment

At 3 months after surgery, patients who participated in leisure activities constituted the leisure activity group, and patients who did not participate in leisure activities constituted the non-leisure activity group. Leisure activity was defined as any activity for the patient’s own pleasure other than housework during leisure time (e.g., walking, light exercise, playing the piano). It was evaluated by interviewing the patients.

### 2.7. Statistical Analysis

A comparison of patient’s participation in leisure activity before and 3 months after surgery was examined using chi-squared test. The difference in QOL before and 3 months after surgery between the leisure activity group and the non-leisure activity group was analyzed using Mann–Whitney U test. Changes in QOL before and 3 months after surgery in the leisure activity group and the non-leisure activity group were also analyzed using Wilcoxon signed-rank test.

The factors of QOL at 3 months after surgery between the leisure activity group and the non-leisure activity group were analyzed. Univariate analysis was performed using Student’s *t*-test, chi-squared test, and Mann–Whitney U test to identify factors associated with patient’s participation in leisure activity 3 months after surgery. Next, including factors with a statistically significant difference of *p* < 0.1 on univariate analysis, logistic regression analysis was used to identify the best independent predictor of participation in leisure activities 3 months after surgery. All statistical analyses were performed using SPSS software version 22.0 (IBM, Tokyo, Japan). A value of *p* < 0.05 was considered statistically significant, and all tests were two-sided.

## 3. Results

### 3.1. Time Course of Quality of Life

Of the 160 patients, 37 were categorized into the leisure activity group and 123 into the non-leisure activity group. Twenty-three patients took part in exercise, eight patients played an instrument, and six patients had other hobbies. Among patients who participated in leisure activities before surgery, 29 were in the leisure activity group at 3 months after the surgery (Table 1). There was a significant decrease in the number of patients who took part in leisure activities at 3 months after surgery compared with before surgery (*p* < 0.05).

### 3.2. Relationship between Quality of Life and Leisure Activity

The differences in QOL before and 3 months after surgery between the leisure activity group and the non-leisure activity group are shown in Table 2. Physical function was significantly different between the leisure activity group and the non-leisure activity group before surgery (*p <* 0.05). Global health status, emotional function, social function, and dyspnea were significantly different between the leisure activity group and the non-leisure activity group at 3 months after surgery (*p* < 0.05) (Table 2). The data of the leisure activity group 3 months after surgery show the improvement in global health status, emotional function, and social function and decrease in physical function (*p* < 0.05) (Table 2). The data of the non-leisure activity group 3 months after surgery show the improvement in emotional function and financial difficulties and decrease in physical function (*p* < 0.05) (Table 2).

### 3.3. Factors Affecting Participation in Leisure Activities at 3 Months after Surgery

The results of the univariate analysis are shown in Table 3. Participation in leisure activities before surgery, BMI, and shoulder abduction ROM-T were significantly different between the two groups (*p* < 0.1). Logistic regression analysis of these three variables showed that only participation in leisure activities before surgery was significantly associated with participation in leisure activities at 3 months after surgery (*p* < 0.05) (Table 4).

## 4. Discussion

Factors related to early postoperative participation in leisure activities at 3 months after surgery were examined in patients after breast cancer surgery. The results showed that preoperative patient’s participation in leisure activity was strongly associated with participation in leisure activities 3 months after surgery.

In our study, the proportion of patients involved in leisure activities was 34% (55 patients) before surgery and 23% (37 patients) at 3 months after surgery, showing a significant difference. The results of this study cannot be compared with other studies because there are no reports examining patient’s participation in leisure activity in the early postoperative period. In this study, however, preoperative and postoperative rehabilitation was provided to patients during hospitalization. Patients were evaluated every month for up to 3 months after surgery. Daily life guidance, including the guidance on leisure activities, was provided according to the results of the evaluation. The number of patients participating in leisure activities may be further reduced when there is no progressive rehabilitation during hospitalization and regular follow-up after discharge. Therefore, postoperative patients with breast cancer require follow-up, including regular rehabilitation after discharge.

In a study of community-dwelling people, sufficient sleep (7–8 h/day) and having a hobby were associated with increased health-related QOL scores [14]. In the present study, global health, emotional function, and social function were significantly higher in the leisure activity group than in the non-leisure activity group. Severe stress was significantly associated with a decrease in health-related QOL scores, and patients who participated in leisure activities may have had a higher QOL because the leisure activities reduced stress. Additionally, participation in leisure activities may be a means of connecting with people, which has a positive effect on QOL. In the comparison between before and 3 months after surgery, the leisure activity group showed improvement in global health status, emotional function, and social function, and patients’ leisure participation was found to have a positive effect on QOL.

Among patients who participated in leisure activities before surgery, 52.7% took part in leisure activities at 3 months after surgery. The factor affecting participation in leisure activities at 3 months after surgery was the presence or absence of involvement in leisure activities before surgery, and patients who participated in leisure activities before surgery were more likely to partake in leisure activities at 3 months after surgery. Active exercise in breast cancer patients does not increase the risk or severity of lymphedema and improves the muscle strength of the limbs, thereby improving QOL and reducing the stress of daily activities [15,16,17,18]. In this study, we encouraged hobbies after surgery and recommended physical exercise and leisure activities. Patients who took part in leisure activities before surgery were able to gain the understanding of their families and receive assistance with achieving free time in which to perform leisure activities, and the required mindset was already present in the patient’s life. Therefore, the patients were able to undertake leisure activities with the reassurance that use of the upper limbs is not harmful after surgery. However, patients who did not participate in leisure activities before surgery were less likely to participate 3 months after surgery, and thus, such individuals require interventions to encourage their involvement. For patients with no particular leisure activities before surgery, medical staff may also be encouraged to share the benefit of performing leisure activities and present available activities according to their condition.

Several arm morbidity variables were significant predictors of difficulties in the participation of recreational activities in patients with breast cancer after surgery [11]. In this study, ROM and upper limb function had no effect on leisure activity involvement. Patients taking part in leisure activities do so according to upper limb function, but ROM and upper limb function may not have affected their participation. Therefore, exercise therapy aimed at improving ROM and upper limb function alone may not be sufficient to encourage patient’s participation in leisure activity.

Breast cancer survivors who are single, divorced, or widowed prefer to return to their work [19,20]. A study has reported a significant association between unemployment and childlessness [21]. In this study, the presence or absence of married status, the presence of children, and the presence of coresident household members did not significantly affect participation in leisure activities at 3 months after surgery. In addition, in this study, there was no relation between returning to work and leisure activity involvement at 3 months after surgery. Partaking in leisure activities was less affected by family circumstances, unlike returning to work. However, other studies reported that frequent community activity such as sports, exercise, leisure activity, or volunteering was significantly lower in partners with functional disabilities than in partners without functional disabilities [22]. It is not clear whether family status affects participation in leisure activities because this study did not investigate the health status of the patients’ families.

### Study Limitations

There are several limitations in this study. First, the number of patients in the leisure activity group was small. Second, differing amounts and frequencies of leisure activity may constitute different factors, but our research could not investigate this. Third, our study was a short-term survey evaluating participation in leisure activities for up to 3 months after surgery, and participation in leisure activities in the long term has not been investigated.

## 5. Conclusions

The purpose of this study was to investigate the factors related to patient’s participation in leisure activity in breast cancer patients with axillary lymph node dissection at 3 months after surgery. The results showed that preoperative patient’s participation in leisure activity was strongly associated with participation in leisure activities 3 months after surgery. To encourage participation in postoperative leisure activities, it is necessary to evaluate the preoperative status of participation; in particular, medical workers need to focus on patients who have not participated.

## Figures and Tables

**Table 1:** Changes between leisure activity group and non-leisure activity group.

Groups	Before	1 Month	2 Months	3 Months
Leisure activity group (yes/no)	29/ 8	27/ 10	34/ 3	37/0
Non-leisure activity group (yes/no)	26/ 97	16/ 107	12/ 111	0/123

Yes: patients who did participate in leisure activities. No: patients who did not participate in leisure activities.

**Table 2:** Comparison of quality of life between the leisure activity group and the non-leisure activity group.

Variable	Leisure Activity Group			Non-Leisure Activity Group			*p*-Value between Groups	
	Before	3 months	*p*-value ^(a)^	Before	3 months	*p*-value ^(b)^	*p*-value ^(c)^	*p*-value ^(d)^
GHS	66.7(8–100)	83.33 (10–100)	0.022	66.7(17–100)	66.66 (8.3–100)	0.373	0.814	0.015
Functional scales, scores								
Physical function	100(33–100)	93.33 (53.3–100)	0.026	93.3(20–100)	86.66 (26.7–100)	0.038	0.046	0.578
Role function	100(0–100)	100 (33.3–100)	0.072	100(0–100)	83.33 (0–100)	0.030	0.203	0.381
Emotional function	75(0–100)	100 (33.3–100)	*p* < 0.0001	75(17–100)	87.495 (16.7–100)	*p* < 0.0001	0.577	0.019
Cognitive function	100(50–100)	100 (50–100)	0.278	83.3(17–100)	83.33 (16.7–100)	0.742	0.328	0.069
Social function	100(0–100)	100 (33.3–100)	0.016	83.3(16.7–100)	91.665 (0–100)	0.850	0.414	0.017
Symptom scales, scores								
Fatigue	22.2(0–100)	22.22 (0–66.7)	0.592	22.2(0–88.9)	33.33 (0–100)	0.146	0.529	0.400
Nausea/vomiting	0(0–100)	0 (0–100)	0.206	0(0–33.3)	0 (0–83.3)	0.050	0.778	0.601
Pain	16.7(0–100)	16.66 (0–66.7)	0.594	16.7(0–83.3)	16.66 (0–100)	0.349	0.422	0.717
Dyspnea	0(0–66.7)	0 (0–100)	0.850	0(0–83.4)	0 (0–100)	0.309	0.582	0.018
Insomnia/sleep	0(0–100)	0 (0–100)	0.617	0(0–100)	33.33 (0–100)	0.574	0.290	0.459
Appetite loss	0(0–66.7)	0 (0–100)	0.670	0(0–100)	0 (0–100)	0.686	0.528	0.916
Constipation	0(0–100)	0 (0–100)	0.868	0(0–66.7)	0 (0–100)	0.293	0.286	0.300
Diarrhea	0(0–100)	0 (0–100)	0.705	0(0–66.7)	0 (0–66.7)	0.543	0.086	0.276
Financial difficulties	0(0–100)	0 (0–100)	0.289	33.3(0–100)	0 (0–100)	0.002	0.187	0.178

Median (minimum–maximum); GHS, global health status. ^(a)^ Comparison of QOL before and 3 months after surgery in the leisure activity group. ^(b)^ Comparison of QOL before and 3 months after surgery in the non-leisure activity group. ^(c)^ Comparison of QOL before surgery between the leisure activity group and the non-leisure activity group. ^(d)^ Comparison of QOL 3 months after surgery between the leisure activity group and the non-leisure activity group.

**Table 3:** Comparison of variables between the leisure activity group and the non-leisure activity group.

Variable	Leisure Activity Group	Non-Leisure Activity Group	*p*-Value
Age, years ^(a)^	54.4 ± 12.5	56.4 ± 11.4	0.375
BMI, kg/m^2 (b)^	21.6(16.8–33.1)	22.5(16.7–34.4)	0.083
Work, yes/no ^(c)^	10/27	28/95	0.660
Level of lymph node dissection, level 1/level 2 and higher ^(c)^	33/4	93/30	0.107
Married, yes/no ^(c)^	29/8	104/19	0.453
Children, yes/no ^(c)^	26/11	97/26	0.276
Coresident household members, yes/no ^(c)^	32/5	110/13	0.567
Neoadjuvant chemotherapy, yes/no ^(c)^	9/28	38/85	0.539
Postoperative chemotherapy, yes/no ^(c)^	18/19	61/62	1.000
Postoperative hormonal therapy, yes/no ^(c)^	8/29	25/98	0.821
Postoperative radiotherapy, yes/no ^(c)^	11/26	44/79	0.558
Shoulder flexion ROM-T, degrees ^(b)^	160(130–175)	155(100–180)	0.194
Shoulder abduction ROM-T, degrees ^(b)^	160(125–175)	155(70–180)	0.075
DASH, scores ^(b)^	12.05(0–36.66)	14.16(0–60)	0.362
Leisure activity participation before surgery, yes/no ^(c)^	29/8	26/97	*p* < 0.0001

^(a)^ Mean ± standard deviation, ^(b)^ median (minimum–maximum), ^(c)^ proportion. BMI, body mass index; ROM-T, range of motion test; DASH, Disabilities of the Arm, Shoulder and Hand.

**Table 4:** Predictors of participation in leisure activity on logistic regression analysis.

Variable	Odds Ratio (95% CI)	*p*-Value
BMI	0.894 (0.785–1.018)	0.090
Shoulder abduction ROM-T	1.017 (0.985–1.050)	0.110
Leisure activity participation before surgery	16.300 (6.198–42.869)	*p* < 0.0001

CI: confidence interval; BMI, body mass index; ROM-T, range of motion test.

## Data Availability

The data presented in this study are available on request from the corresponding author. To protect participants’ personal information, the data are not publicly available.
